# Harnessing Artificial Intelligence for Qualitative and Mixed Methods in Nursing Research

**DOI:** 10.7759/cureus.48570

**Published:** 2023-11-09

**Authors:** Abdulqadir J Nashwan, Hana Abukhadijah

**Affiliations:** 1 Nursing Department, Hamad Medical Corporation, Doha, QAT; 2 Epidemiology and Public Health Department, Hamad Medical Corporation, Doha, QAT

**Keywords:** nursing research, large language models, artificial intelligence, mixed-methods, qualitative studies

## Abstract

This editorial discusses the transformative potential of artificial intelligence (AI), particularly large language models (LLMs), in enhancing traditional nursing research methodologies, specifically qualitative and mixed methods. The article emphasizes the benefits of AI such as LLMs in data processing, analysis, integration, and triangulation while also underscoring the importance of addressing ethical concerns and the need for proper training for researchers.

## Editorial

Nursing research is pivotal to advancing evidence-based practice (EBP), enhancing patient outcomes, and ensuring optimal care delivery [1]. Historically, nursing research - rooted in social science - has relied heavily on qualitative and mixed methods (MM), emphasizing the value of human experiences and narratives and the contextual nuances of patient care [2]. With the evolution and progression of artificial intelligence (AI), there is an unprecedented potential to revolutionize these traditional methodologies.

Qualitative research involves in-depth interviews, focus groups, and observational data. The vast amount of narrative data collected can be overwhelming, making the analysis process time-consuming and sometimes subjective [3]. Advanced AI tools can swiftly transcribe audio and video recordings, making it feasible to process large datasets. Furthermore, with globalized nursing research, translation capabilities ensure that language is no longer a barrier. Natural language processing (NLP), a subfield of AI, can be harnessed to identify patterns, themes, and sentiments in textual data, augmenting the researcher's capacity to grasp underlying meanings and draw more robust conclusions [4].

On the other hand, MM, which combines qualitative and quantitative approaches [5], benefits from AI's ability to discern complex patterns. For instance, while a nurse researcher might notice broad trends in patient narratives about pain experiences, AI can help identify subtle, recurrent patterns or themes across more data - potentially unveiling previously unnoticed insights about pain management or patient experiences. Moreover, data interpretation in MM requires integrating numeric and thematic data [6]. AI-powered data visualization tools can assist in merging these data types, offering interactive visuals that provide deeper insights and aid in effectively communicating research findings.

One core principle of qualitative nursing research is understanding the patient's story or lived experience [7]. Utilizing AI can help gather a broader range of narratives from informants. Integrating AI-driven platforms like Chatbots with electronic health records can enable patient diaries, feedback, and real-time symptom tracking. This ensures that the patient's perspective is always included in research.

Harnessing AI in qualitative and MM nursing research is challenging. Concerns around data privacy, the de-identification of patient narratives, and potential biases in AI algorithms are paramount [8,9]. Therefore, alongside the integration of AI, there is a pressing need to constantly refine and update ethical guidelines, ensuring the technology is used responsibly and respects the dignity and rights of all informants.

The global nursing community can benefit from AI's ability to bridge gaps [10]. AI-powered platforms can facilitate collaboration, allowing researchers from different regions to co-analyze data, share insights, and jointly contribute to global nursing knowledge [10]. In nursing research, there is always a window for improvement [10]. By continually integrating the latest AI tools and methodologies, nursing researchers can ensure that their methods remain cutting-edge, effective, and relevant.

Recent AI development has sparked enthusiasm for its potential use in research. The nursing profession is being transformed by AI [11], so nurse leaders must be visionaries and think genuinely about the implications of AI. This editorial provides an unprecedented view of exploring AI applications in nursing qualitative research and MM.

This paper discusses how AI tools can mimic human researchers, thus increasing the efficiency of qualitative analysis. It is worth noting that AI in qualitative research is a complementary tool rather than a replacement for human researchers, and the results of AI-assisted analysis should be interpreted and contextualized with human judgment and expertise. Here, we will explore some examples of AI-assisted qualitative and MM analysis:

Step 1: transcribing interviews/videos

A transcription service that leverages AI tools or deep learning models, such as ChatGPT (Chat Generative Pre-trained Transformer; OpenAI, Inc., Delaware) represents a revolutionary advancement in the realm of qualitative research. Unlike traditional manual transcription methods, which can be time-consuming and prone to human error, these AI-powered services can rapidly process hours of audio or video recordings with remarkable precision. By swiftly converting spoken content into written text, researchers can expedite the onset of their qualitative data analysis. Moreover, the sophistication of these models allows them to recognize and differentiate between various speakers, dialects, and even nuanced linguistic nuances, which can be crucial for ensuring the accuracy and integrity of the transcribed content. The efficiency brought about by such systems means that researchers can spend more time on in-depth analysis and interpretation of their data rather than getting bogged down in the initial transcription phase. Additionally, AI-driven transcription services can be continuously updated and refined, ensuring that they evolve and improve, adapting to new languages, accents, and terminologies. Therefore, by integrating AI into transcription, the process is made faster and more efficient, and the quality and reliability of the transcriptions are elevated, allowing researchers to derive richer insights from their qualitative data.

Step 2: generating a general summary of the text

Using structured ChatGPT prompts can summarize lengthy interviews so you can have brief information about each participant and understand them before analyzing the data. Structured prompts feed the system with adequate information to get what you want to tell the system to look at the transcript and instruct the tool that every answer should be based on the text you provided. For example, the ChatGPT prompt: "Summarize the transcript in 1000 words: (Text)" will summarize and inform you about your interviewee before you go line by line and manually develop code to address your research question.

Step 3: open coding

The AI tool can support the researcher by generating initial codes and a summary that addresses the research question using the prompt. For example, the ChatGPT prompt: "Act as a researcher. I will now upload an interview transcript for you to code based on the research question, known as qualitative, initial, or open coding. The questions asked by the interviewer do not need to be coded. The codes must be detailed and descriptive. Review the transcript to identify relevant excerpts that address the research question (RQ: here) and generate codes that best represent the excerpts identified; each code should be between two to five words. An interview transcript is provided in the text "text"."

Step 4: develop themes

Utilizing AI tools for coding qualitative data presents a transformative approach for researchers. Instead of manually sifting through vast amounts of text to identify patterns and themes, AI systems like ChatGPT can quickly scan the data and suggest potential categories. These suggestions are based on the AI's contextual understanding and pattern recognition capabilities, which can discern recurring topics or sentiments within the text. By employing AI in this manner, researchers can ensure a more objective and consistent coding process. Additionally, this automated approach can highlight unforeseen themes that might be overlooked in manual analysis. When ChatGPT, for instance, is tasked to categorize and code, it utilizes its extensive training data to offer relevant themes, ensuring a comprehensive and nuanced understanding of the qualitative content. This integration of AI not only streamlines the analysis process but also enhances the depth and breadth of insights derived.

Step 5: synthesis and visualization of qualitative data

AI's capability to generate thematic maps or word clouds revolutionizes how researchers visualize qualitative data. By transforming raw text into visual representations, the data becomes more accessible and comprehensible. These graphical illustrations allow for immediate recognition of dominant themes or frequently mentioned terms, enabling researchers to rapidly grasp their findings' essence. When platforms like ChatGPT are tasked with such operations, they can sift through vast amounts of data and highlight key themes, subsequently organizing them into tables or charts. For instance, a table generated by ChatGPT might have one column listing dominant themes and an adjacent column showcasing representative excerpts from the transcript. This provides a clear, structured overview of the findings and offers researchers direct quotes that encapsulate the essence of each theme, thereby enriching their analysis and presentation of results.

Step 6: triangulation of results for MM designs

**Figure 1:**
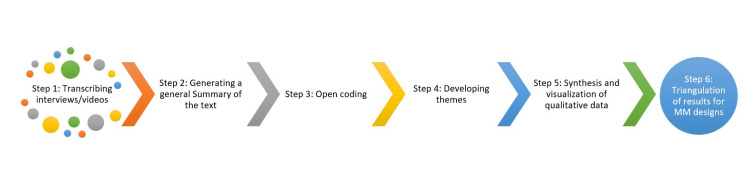
Summary of the six-step process for integrating LLMs into qualitative and mixed methods research large language models (LLMs); mixed methods (MM) Image Credits: Abdulqadir J. Nashwan

Triangulation gathers complementary yet different data for specific research topics to enhance validity and credibility [12]. For example, ChatGPT can assist in the triangulation of results for MM research designs by aiding in literature reviews, offering guidance on quantitative data analysis, such as descriptive statistics and test selection, and advising on qualitative data analysis techniques like coding and thematic analysis [13]. Additionally, it can help in the integration of qualitative and quantitative results, providing methodological insights and assisting in critical evaluation [13]. ChatGPT can also guide the drafting and writing of findings, addressing post-analysis questions [13]. Figure 1 summarizes the six-step process for integrating LLMs into qualitative and mixed-methods research.

Conclusion

AI has transformative potential for qualitative and MM nursing research. AI can amplify the depth and breadth of insights derived by enhancing data collection, analysis, and interpretation. Nevertheless, as with any new technology, its application requires careful consideration, skillful handling, and continuous development. As nursing researchers venture deeper into AI, the balance between technological prowess and the human touch becomes pivotal. In this harmonized approach, the future of nursing research looks promising, with the potential to significantly improve patient care and the broader healthcare landscape.
